# Pacemaker Failure Due to Loss of Fluid Seal in a Patient with a 5/6mm Pacemaker Header Port and a 5mm Unipolar Lead

**DOI:** 10.1016/s0972-6292(16)30691-x

**Published:** 2013-11-15

**Authors:** Paul Chun Yih Lim, Boon Yew Tan, Kah Leng Ho, Chee Keong Ching, Wee Siong Teo

**Affiliations:** Department of Cardiology, National Heart Centre Singapore

**Keywords:** 5/6 mm, pacemaker failure, blood leak

## Abstract

**Introduction:**

Before IS-1 (3.2 mm) standardization of pacemaker leads and connectors, 5/6 mm connector ports accomodated 5mm or 6mm diameter lead connector pins.

**Case report:**

A patient with sick sinus syndrome underwent implantation of a 5mm unipolar atrial lead, mated to a 5/6mm connector port Medtronic Spectrax Sx 5985 pacemaker. Pulse generator reached ERI in 2006, with change out to a Medtronic Sigma SSR306 (5/6mm connector port) and preservation of the 5mm lead. She was admitted in 2010 for atrial lead non capture from blood leak and corrosion of the header-connector pin apparatus.

**Discussion:**

5/6mm pacemaker header ports have a 5mm flexible sealing ring at the port entrance to seal 5mm or 6mm lead connector pins. The inner barrel diameter of the connector port is 6mm and insertion of a 5mm lead results in a 0.5mm tolerance circumferentially. Should the seal be compromised, blood can corrode the apparatus. To minimize this, we can employ (a) a cinching tie to further seal the silicone ring (b) universal adaptor sleeves (c) splice kits (d) lead adaptor kits. Aging leads, adaptor kits or sleeves themselves can result in lead failure. It may be safer to re-implant the entire system.

**Conclusions:**

A 5/6mm configuration pacemaker header connector port allows for significant tolerances when a 5 mm lead is used. Consideration must be made to prevent leaks.

## Introduction

Before industry-wide IS-1 (3.2 mm) standardization of pacemaker lead and connectors in the 1980s, 5mm or 6mm unipolar connector pins for permanent pacemaker leads were common. To accommodate this, connector ports which had the 5/6mm configuration were made available. However, after IS-1 standard pacemakers and leads were introduced, pulse generator changes for patients with previous 5/6mm leads and header ports have become increasingly rare. We report a case of pacemaker dysfunction in an 81 year old patient arising from blood leak and subsequent corrosion of the connector block as a result of the 5/6mm connector port combination with a 5mm lead connector pin, and further discuss the options for management strategies for patients with prior 5/6mm leads and header ports.

## Clinical presentation

**Figure 1:**
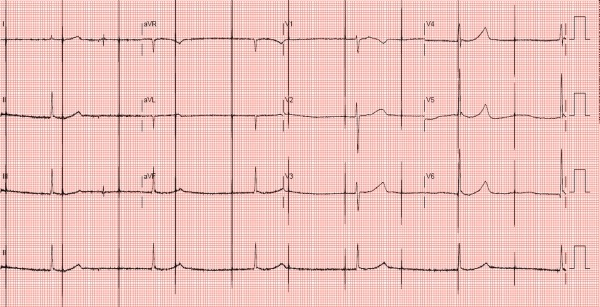
12 lead electrocardiogram on admission shows narrow complex junctional escape rhythm with no obvious P waves. Unipolar atrial pacing spikes seen at rate of 60 per minute with no evidence of atrial capture.

An 81 year old female with sick sinus syndrome was admitted with near syncopal events in September 2010. She had underwent implantation of a 5mm Medtronic 4057 unipolar active fixation atrial lead, mated to a 5/6mm connector port configuration Medtronic Spectrax Sx 5985 single chamber pacemaker in 1990. Pulse generator ERI reached in 2006, and a Medtronic Sigma SSR306 (which also had the 5/6mm connector port configuration) was implanted, with preservation of the previous 5mm lead. She had been well since the pulse generator change out. Her electrocardiogram on this admission showed intermittent symptomatic non capture of the atrial lead and pauses (Figure 1). Pacemaker interrogation however, showed normal lead function. Chest radiograph was also normal.

**Figure 2:**
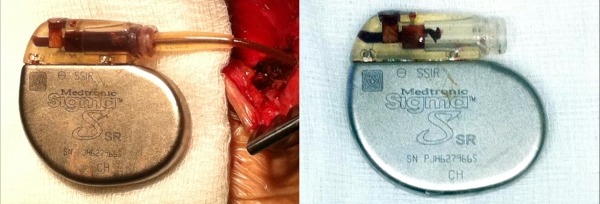
Explanted Medtronic Sigma SSR306 pulse generator showing blood leak and clot within the header (left) and with the 5mm Medtronic 4057 unipolar lead removed showing extensive corrosion in the set –screw connector block (right).

She underwent a pacemaker system revision on the 22nd of September 2010. Blood leak (and clot formation) into the pulse generator header beyond the silicone barrier ring was immediately evident. The fixation screw apparatus showed no evidence of leak through the screw hole. There was also evidence of severe corrosion of the connector port and lead connector. (Figure 2). The atrial lead was non-salvageable and hence capped and abandoned. A new IS-1 right ventricular lead was inserted and a Medtronic Relia RES01 pulse generator was implanted.

## Discussion

Ever since the establishment of IS-1 standards, patients implanted with non IS-1 standard devices and leads, though becoming progressively rarer, still do present for pulse generator change outs or upgrades. Based on statistics from our centre, out of 337 device related procedures in 2009 (consisting of device and lead implants/revisions/changes), 59 were pacemaker pulse generator changes, of which 5 consisted of 5 or 6mm lead and header systems. In 2010, out o f 367 device related procedures (including 36 pulse generator changes), only 1 (0.3%) was for a non IS-1 system. The next year, only 1 non IS-1 pulse generator change was performed at our centre (out of 46 pulse generator changes contributing to 389 device related procedures) (0.3%). Though numbers are progressively fewer, cardiologists, especially those possessing minimal experience with older 5 or 6mm systems, may still encounter these patients. Hence, in this discussion, we will describe the design of these 5/6mm header ports and aim to bring up pertinent points regarding management options for such patients.

**Figure 3:**
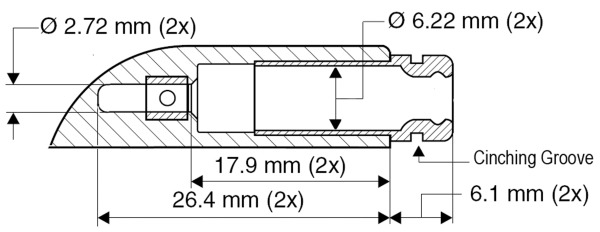
Diagram of the 5/6mm header with a 6.22mm inner barrel diameter and an outer flexible silicone ring which can accommodate a 5mm or 6mm lead. A cinching groove is also evident around the silicone ring.

Pacemaker header connector port in the 5/6mm configuration is designed to accommodate both the 5 mm and 6 mm lead pin variations. This is because the 5/6mm connector port has a silicone sealing ring at the entrance of the port which is sufficiently flexible to accommodate both leads. However, the inner diameter of the barrel of the connector port is 6mm. While a 6 mm lead would fit perfectly, a 5mm lead would have a 0.5mm tolerance circumferentially (Figure 3). Should the silicone sealing ring at the entrance of the header become compromised, then it is conceivable (as in this case) for bodily fluids to migrate to the connector pins and erode them. Furthermore, in the situation where the lead curves off to an angle immediately after the exit point of the silicone sealing ring and does not enter the apparatus exactly parallel to the header port, (due to short leads or coiled leads) this leads to poor strain relief at the tip of the silicone ring and may contribute to the failure of the seal.

To minimize this, a cinching tie can be applied around the groove at the lead entrance of the header, especially if a 5/6mm connector port is mated with a smaller 5 mm lead (Figure 3). This extra step was unfortunately not performed during the pulse generator change out in 2006. Blood leak and the resultant connector pin erosion with intermittent short in the header likely accounted for the intermittent loss of capture in the atrial lead.

Alternative approaches in overcoming the 5/6mm header and 5mm lead mismatch include: (a) utilization of a universal adaptor sleeve to upsize the 5mm lead connector to 6mm in diameter, and (b) utilization of medical grade silicone sleeves with medical adhesives to increase lead diameter (splice kits).[1] There is also the Medtronic Model 5866-37 M lead adaptor kit, which is designed to connect a 5 mm unipolar lead to an IS-1 unipolar connector block pulse generator.

Adaptor kits such as the Model 5866-37 M requires the use of setscrews to anchor the old lead, while sleeves require adhesives to upsize the 5 mm lead. The added components in these systems may introduce another element for lead failure. In a study in 1994, 8 out of 14 adaptor kit 366-08 used failed during an average follow up of just 32.1 months.[2] Aging leads also have higher failure rates, an important consideration, especially if a decision is made to preserve these leads. Added to this is operator experience in using adopter kits and sleeves, or simply the need for a cinching anchor during change out. Ultimately it maybe simpler, and safer, to either extract or abandon the older lead and re-implant the entire system.

## Conclusions

A 5/6mm configuration pacemaker header connector port allows for significant tolerances when a 5 mm lead is used. Consideration must be made for the use of an adaptor kit, upsizing sleeves or a cinching tie to avoid bodily fluid leaks into the header. Otherwise, reimplantation of a newer IS-1 lead and pulse generator may be a safer option.
